# Neuropathology of wild-type and *nef*-attenuated T cell tropic simian immunodeficiency virus (SIVmac32H) and macrophage tropic neurovirulent SIVmac17E-Fr in cynomolgus macaques

**DOI:** 10.1007/s13365-012-0084-3

**Published:** 2012-03-09

**Authors:** Sean Clarke, Neil Berry, Claire Ham, Jack Alden, Neil Almond, Debbie Ferguson

**Affiliations:** Division of Retrovirology, National Institute of Biological Standards and Control, Blanche Lane, South Mimms, Hertfordshire, EN6 3QG UK

**Keywords:** SIV, Neuropathology, Cynomolgus macaques, SIVmacJ5, SIVmacC8, SIVmac17E-Fr

## Abstract

The neuropathology of simian immunodeficiency (SIV) infection in cynomolgus macaques (*Macaca fascicularis*) was investigated following infection with either T cell tropic SIVmacJ5, SIVmacC8 or macrophage tropic SIVmac17E-Fr. Formalin fixed, paraffin embedded brain tissue sections were analysed using a combination of in situ techniques. Macaques infected with either wild-type SIVmacJ5 or neurovirulent SIVmac17E-Fr showed evidence of neuronal dephosphorylation, loss of oligodendrocyte and CCR5 staining, lack of microglial MHC II expression, infiltration by CD4^+^ and CD8^+^ T cells and mild astrocytosis. SIVmacJ5-infected animals exhibited activation of microglia whilst those infected with SIVmac17E-Fr demonstrated a loss of microglia staining. These results are suggestive of impaired central nervous system (CNS) physiology. Furthermore, infiltration by T cells into the brain parenchyma indicated disruption of the blood brain barrier (BBB). Animals infected with the Δ*nef*-attenuated SIVmacC8 showed microglial activation and astrogliosis indicative of an inflammatory response, lack of MHC II and CCR5 staining and infiltration by CD8^+^ T cells. These results demonstrate that the SIV infection of cynomolgus macaque can be used as a model to replicate the range of CNS pathologies observed following HIV infection of humans and to investigate the pathogenesis of HIV associated neuropathology.

## Introduction

Neuropathology and symptoms of central nervous disease are late complications of HIV-1 infection and before widespread use of antiretroviral therapies the most severe form, HIV-1 associated dementia (HAD), developed in 20–30% of patients (Gonzalez-Scarano and Marin-Garcia 2005; Kaul et al. 2005). Despite the use of continually advancing drug therapy regimes over the last two decades up to 40% of HIV-infected individuals still develop a range of HIV-1 associated neurocognitive disorders (HAND; Antinori et al. 2007) that can greatly affect daily life and potentially disrupt adherence to their drug regime.

HIV rapidly enters the central nervous system following infection, probably transiting the blood brain barrier within infected monocytes and lymphocytes (Liu et al. 2002; Albright et al. 2003). The exact cause of the ensuing neuronal damage is currently unknown but the potential exists for both viral proteins and/or the resulting inflammatory response to be the causative agent (Gonzalez-Scarano and Marin-Garcia 2005; Kaul et al. 2005).

A pathology of HIV encephalitis (HIVE) can develop, characterised by the presence of astrogliosis, microglial nodules, accumulation of perivascular macrophages and multinucleated giant cells (MNGC; Flaherty et al. 1997; Overholser et al. 2003) and may be present despite very low levels of viral replication within the CNS (Valcour et al. 2011).

Despite the re-definition of the research diagnostic categories of HAND in 2007 (Antinori et al. 2007) variability in how neuropsychological testing is performed in a busy or under resourced clinical setting makes it difficult to follow the development of HAND on an epidemiological scale. It is therefore still a matter for debate whether the categories of HAND define a pathway along which patients will inevitable progress or whether they define distinct types of neurocognitive disorders, the development of which are determined by a combination of viral and host factors.

The experimental infection of macaque species with strains of SIV has been used for a number of years to investigate pathology associated with HIV infection. Many of the pathological characteristics observed in HAD and HIVE have been found within SIV-infected macaque brains (Desrosiers and Ringler 1989; Ringler et al. 1998; Sharer et al. 1988). However, much of this work has been undertaken using a combination of macaque species and macrophage tropic virus strains designed to result in the rapid development of disease and neurological pathology. For example, a model developed by Zink et al. using pig-tailed macaques (*Macaca nemestrina*) co-infected with SIVmac17E-Fr and SIVmacDeltaB670 (Clements et al. 2002; Craig et al. 1997; Zink et al. 1999) was developed to ensure that the maximal numbers of infected animals developed rapid SIV-related neurological complications. Whilst these models are valuable to investigate late stage neurological disease pathology and to evaluate potential therapeutic interventions, rapid disease development is not the prevalent phenotype in humans (Williams et al. 2008). Furthermore, they are not necessarily appropriate for research to establish the pathogenesis of neuropathology. The availability of a model exhibiting a more gradual disease development may prove more suitable for investigating the early events that follow SIV infection and identify opportunities for novel prophylactic treatments that specifically prevent or control neuropathology.

Compared with rhesus (*Macaca mulatta*) and pig tail macaques, more limited information is available about the ability of SIV to enter and cause pathology within the CNS of cynomolgus macaques. The development of neurovirulent lesions in small-scale studies has been reported following infection with either the SIVmac17E-Fr-SIV/DeltaB670 combination (Zink et al. 1997) or various SIVsmm strains (Baskerville et al. 1990; Li et al. 1991). Neuroinvasion of the macrophage tropic SIVmac251 32H was associated with a progressive loss of neuronal diameter (Montgomery et al. 1998) and progressive dendritic pathology (Montgomery et al. 1999) whereas infection with the lymphotropic SIVmac239 resulted in an increase in neuronal diameter (Montgomery et al. et al. 1998). Infection with neither virus has been associated with the formation of an MNGC pathology within the CNS, as is frequently reported in the more pathogenic rhesus and pig-tail macaque models.

To understand the neuropathological consequences of SIV infection of cynomolgus macaques and its relationship with peripheral markers of viral replication, we undertook a comparative study of CNS tissue collected from three groups of cynomolgus macaques patently infected with either T cell tropic wild-type SIVmacJ5 (Rud et al. 1994), Δ*nef* attenuated SIVmacC8 (Rud et al. 1994) or with macrophage tropic SIVmac17E-Fr (Flaherty et al. 1997). Evidence of SIV infection was detected in samples of brain from all macaques analysed. However, distinct patterns of neuropathology were associated with each virus. These data indicate that SIV enters the brains of cynomolgus macaques even when there is limited viral infection detectable in the blood. These models may be valuable to study SIV neuropathological events that occur prior to or in the absence of clinical neurological disease and following infection with SIV strains that are representative of a range of peripheral viral replication levels.

## Materials and methods

### Animals

Purpose bred juvenile, SRV-ve and STLV-ve cynomolgus macaques were used in these studies. The animals were housed and maintained in accordance with Home Office guidelines for care and maintenance of non-human primates. Animals were sedated with ketamine hydrochloride (Vetalar V, Pharmacia Animal Health Ltd, Corby, UK) before inoculation of virus or sampling of blood by venepuncture. On each of these occasions, sedated macaques were weighed and inspected for general health and signs of disease. At the end of the study, macaques were killed humanely by an overdose of anaesthetic.

### Virus

SIVmacJ5 and SIVmacC8 were derived by molecular cloning from the 11/88 pool of SIVmac32H as previously described (Rud et al. 1994). SIVmacC8 is genetically identical with SIVmacJ5 except for an in-frame 4 amino acid deletion in Nef, 2 other conservative amino acid changes in Nef and a small number of nucleotide changes in the U3’ LTR region not associated with currently known functional motifs. SIVmacC8 exhibits an attenuated phenotype in rhesus and cynomolgus macaques (Almond et al. 1995; Cranage et al. 1997; Clarke et al. 2003). SIVmac17E-Fr was derived from SIVmac239 as previously described (Flaherty et al. 1997) and was kindly provided by JE Clements (Johns Hopkins School of Medicine, Baltimore).

### Experimental design

Two groups of four animals were inoculated intravenously with either 5 × 10^3^ TCID_50_ SIVmacC8 (group A, W250-W253) or 5 × 10^3^ TCID_50_ SIVmacJ5 (group B, W254-W257). 20 weeks following this initial inoculation, both groups were challenged with 50 TCID_50_ SIVmac17E-Fr. A third group of SIV naive animals (group C, X69-X72) were also challenged with SIVmac17E-Fr at this point to provide a challenge control group. Groups A, B and C were euthanized 23 weeks after SIVmac17E-Fr challenge and a number of tissue samples including the entire brain and brain stem collected at necropsy for analysis. Equivalent brains taken at necropsy from SIV naïve juvenile macaques were used to establish baseline levels of staining within SIV negative tissues. Groups consisted of animals of either sex.

### Virological assessments

SIV viral RNA loads were determined in plasma samples collected at various times throughout the course of the study by quantitative real-time reverse transcriptase PCR, as described previously (Berry et al. 2008). Differential *nef* specific DNA PCR and restriction endonuclease analyses were performed to distinguish SIVmac17E-Fr from SIVmac J5 or SIVmacC8. Following nested PCR amplification of the *nef* gene SIVmacC8 and SIVmacJ5 were differentiated by Rsa1 digestion (Rose et al. 1995) and a further restriction digest with *Nsi 1* to differentiate SIV17E-Fr from SIVmacC8 and SIVmacJ5

### T cell and platelet analyses

The presence and proportion of CD4^+^ and CD8^+^ cells in peripheral blood was determined by an immunostaining technique involving antibodies directly conjugated to fluorochromes as described previously (Stebbings et al. 2002).

Platelet levels were determined by processing whole peripheral blood through a Beckman Coulter AcT. 5 diff haematology analyser and comparing against Coulter Act. 5 diff Control Plus standard samples.

### Tissue preparation

Whole brains were harvested post mortem and fixed in 10% (*v*/*v*) formal saline for 4 weeks at 4°C. Formalin fixed brains were then dissected into the following sections: frontal lobe, parietal lobe, temporal lobe, occipital lobe, thalamus, midbrain, pons, medulla oblongata and cerebellum prior to embedding in paraffin wax using standard histological procedures. Four micron sections were cut from each embedded brain section and mounted on poly-L lysine coated slides. Prior to any treatment, sections were de-waxed in xylene and re-hydrated in graded ethanol/water solutions.

### In situ hybridisation

In situ hybridisation was carried out using digoxigenin (dig; Roche, Lewes, UK) labelled single stranded DNA probes and the Omnislide hybridisation system (Thermo Fisher Scientific, MA, USA). A cocktail of either three probes normal to or three probes complementary with SIV transcripts were used within the hybridisation mix as described previously (Canto-Nogues et al. 2001). After extensive washing, bound probes were detected using an alkaline phosphatise/BCIP/NBT chromagenic reaction (Roche, Lewes, UK) for 2 h at room temperature (Ferguson et al. 2007). Sections were washed, counter stained with neutral red, air-dried and mounted in Loctite Super Glue (Denton 1987). Quantification of ISH positive cells was performed by manually counting all positive cells within up to 10 random fields of view (×10 lens and ×10 eyepiece magnification; equivalent to 2.2 mm^2^) and data calculated as the mean number of positive cells per mm^2^. Tabulated results reflect the means score of all animals within that group.

### Immunohistochemistry

Immunohistochemical analyses were performed as previously described (Ferguson et al. 2007). Unmasking of antigens to allow binding of the antibody was undertaken by the optimal technique for each combination of antibody and antigen. Sections were incubated in 50 μg mL^−1^ proteinase K (Roche, Lewes, UK) in PBS pH 7.4 for 15 min at 37°C prior to immunolabelling with CD3, CD8 (C8/144B), CD68 (KP1), GFAP (astrocytes), CD163 (GH1/61; Santa Cruz Biotechnology Inc, California, USA) and CCR5 (3A9; Pharmingen, Oxford, UK). Sections were heated at full power (800 W) for 5 min fully immersed in Vector unmasking solution (Vector Laboratories, Peterborough, UK) previously heated to 96°C for immunolabelling of CD4 (H370, Santa Cruz, Autogen Bioclear, Wiltshire), CNPase1 (oligodendrocytes, 11-5B) (Neomarkers), FF1 (phosphorylated neurofilaments, Dr E Gardner, William Paterson University, USA) or 10 mM citrate buffer (pH 6), KK41 (SIV gp41, Kent et al. 1991), KK75 (SIV Nef, Arnold et al. 1999). Sections were treated using a MenaPath pressurised antigen access unit (125°C 30 s, 90°C 10 s, Access Super solution) for immunolabelling with iba-1 (microglia; Menarini Diagnostics). MHC II staining (CR3/34; DAKO, Ely, Cambridgeshire, UK) was performed on a BondMax automated staining module (Leica Microsystems, USA) using a 30-min HIER1 unmasking protocol and Bond Polymer Refine staining system.

All tissue staining was graded by two independent experienced assessors and tabulated results generated for the means score of all animals within that group.

## Results

### Analysis of viral replication in vivo

Following inoculation of group A with SIVmacC8 and group B with SIVmacJ5 SIV RNA was detected in plasma collected at day 14 post challenge (Table 1). The log_10_ mean viral loads were 4.04 ± 0.55 for group A and 5.27 ± 0.66 for group B. Diagnostic PCR on DNA purified from blood collected at this time confirmed that all macaques were infected (Table 1). On the day of inoculation of SIVmac17E-Fr, 20 weeks after inoculation of the first virus, log_10_ mean SIV viral loads were 2.38 ± 0.90 for group A and 2.67 ± 1.32 for group B. At week 22 of the study (14dpc), log_10_ mean viral loads for group A were 2.13 ± 0.7, group B 2.55 ± 1.4 and for group C 4.73 ± 0.43. At week 43 of the study (23wpc), when all macaques were killed humanely, the mean log_10_ viral loads for group A were 1.93 ± 0.83, group B 2.33 ± 1.4 and for group C ≤1.3 ± 0.00

**Table 1:** Viral replication within the periphery following viral challenge and periphery and tissues following termination

													Termination 20 weeks post SIVmac 17E-Fr								
			Blood 14dpc				Blood doc			Blood 14dpc 17E-Fr				DNA PCR							
Group	Animal	Virus	vRNA log_10_	DNA PCR		Virus Wk20	vRNA log_10_	DNA PCR		vRNA log_10_	DNA PCR		vRNA log_10_	Blood		Spleen		MLN		PLN	
		Week 0		C8	J5			C8/J5	17E-Fr		C8/J5	17E-Fr		C8/J5	17E-Fr	C8/J5	17E-Fr	C8/J5	17E-Fr	C8/J5	17E-Fr
A	W250	SIVmacC8	3.30	+	n/a	SIVmac17E-Fr	3.31	+	n/a	3.00	+	−	3.09	+	−	−	−	+	−	+	−
	W251		4.00	+	n/a		2.92	+	n/a	2.15	+	−	1.35	−	−	+	−	+	−	+	−
	W252		4.25	+	n/a		1.99	+	n/a	2.06	+	−	1.98	−	−	+	−	+	−	+	−
	W253		4.60	+	n/a		1.30	+	n/a	−	+	−	1.30	−	−	+	−	+	−	+	−
B	W254	SIVmacJ5	5.20	n/a	+	SIVmac17E-Fr	2.28	+	n/a	2.31	+	−	2.45	+	−	+	−	+	−	+	−
	W255		4.40	n/a	+		2.33	+	n/a	2.11	+	−	−	+	−	+	−	+	−	+	−
	W256		5.49	n/a	+		4.57	+	n/a	4.46	+	−	4.27	+	−	+	−	+	−	+	−
	W257		5.97	n/a	+		1.50	+	n/a	−	+	−	−	+	−	+	−	+	−	+	−
C	X69	–	n/a	n/a		SIVmac17E-Fr	n/a	n/a		4.22	−	+	−	n/a	−	n/a	+	n/a	+	n/a	+
	X70		n/a	n/a			n/a	n/a		4.92	−	+	−	n/a	−	n/a	−	n/a	+	n/a	+
	X71		n/a	n/a			n/a	n/a		4.57	−	+	−	n/a	−	n/a	+	n/a	+	n/a	+
	X72		n/a	n/a			n/a	n/a		5.21	−	+	−	n/a	+	n/a	−	n/a	+	n/a	+

The presence of viral RNA within peripheral blood was determined 14 days post either SIVmacC8 or SIVmacJ5 viral challenge, day of and 14 days post SIVmac17E-Fr challenge and at termination. A DNA PCR was also used at these time points to discriminate between viral species within peripheral blood and tissues. vRNA levels of—below detectable level of log_10_1.30

Differential DNA PCR analysis of blood cells collected following SIVmac17E-Fr challenge detected the presence of SIVmacC8 only in group A, SIVmacJ5 only in group B and SIVmac17E-Fr only in group C macaques.

### Haematological parameters

The proportion of CD3^+^/CD4^+^ and CD3^+^/CD8^+^ T cells and platelet levels (×10^9^/L) were determined at specific times throughout the study. These data are presented in Table 2. Prior to virus challenge the mean CD3^+^/CD4^+^ lymphocytes for group A were 44.1% ± 5.9%, CD3^+^/CD8^+^ lymphocytes were 20.5% ± 2.2% and platelet levels 400 × 10^9^/L ± 70 × 10^9^/L. For group B CD3^+^/CD4^+^ lymphocytes were 44.9% ± 3.5%, CD3^+^/CD8^+^ lymphocytes were 25.9% ± 6.8% and platelet levels 422 × 10^9^/L ± 62 × 10^9^/L. For group A 14dp SIVmacC8 challenge the mean CD3^+^/CD4^+^ lymphocytes were 34.2% ± 10.3% and the mean CD3^+^/CD8^+^ lymphocytes were 23.6% ± 8.8%. For group B 14dp SIVmacJ5 challenge the mean CD3^+^/CD4^+^ lymphocytes were 35.5% ± 4.0% and the mean CD3^+^/CD8^+^ lymphocytes were 32.7% ± 2.1%.

**Table 2:** Percentage levels of CD3^+^CD4^+^ T cells, CD3^+^CD8^+^ T cells and platelets (×10^9^/L) within peripheral blood were determined 14 days post either SIVmacC8 or SIVmacJ5 viral challenge, day of and 14 days post SIVmac17E-Fr challenge and at termination

		Pre Challenge				14 dpc				Day of challenge			14dpc 17E-Fr			Termination 20 week post 17E-Fr		
Group	Animal	%CD3^+^	%CD3^+^	P’lets	Virus	%CD3^+^	%CD3^+^	P’lets	Virus	%CD3^+^	%CD3^+^	P’lets	%CD3^+^	%CD3^+^	P’lets	%CD3^+^	%CD3^+^	P’lets
		CD4^+^	CD8^+^	×10^9^/L	Week 0	CD4^+^	CD8^+^	×10^9^/L	Week 20	CD4^+^	CD8^+^	×10^9^/L	CD4^+^	CD8^+^	×10^9^/L	CD4^+^	CD8^+^	×10^9^/L
A	W250	51.5	19.8	449	SIVmacC8	46.4	23.2	nd	SIVmac 17E-Fr	47.7	19.2	348	50.7	19.9	398	50.8	19.5	362
	W251	37.4	22.2	312		36.9	32.8	nd		34.6	22.2	230	37.6	20.8	nd	38.1	18.1	241
	W252	42.0	17.6	463		21.6	11.8	nd		28.4	9.5	395	15.1	5.7	424	52.2	13.1	379
	W253	45.6	22.3	376		31.8	26.6	nd		30.7	27.9	308	32.8	33.9	300	36.7	26.8	313
B	W254	44.0	35.8	503	SIVmacJ5	35.9	31.7	nd	SIVmac 17E-Fr	36.7	31.9	423	41.8	34.9	400	30.2	47.8	396
	W255	44.6	20.9	416		29.7	34.1	nd		31.2	28.3	429	35.7	22.9	418	37.5	23.7	481
	W256	49.7	24.2	415		38.2	34.8	nd		27.5	30.2	335	39.1	30.8	328	28.4	26.9	69
	W257	41.2	22.4	352		38.2	30.3	nd		40.0	24.4	308	42.5	23.9	nd	39.0	29.3	366
C	X69	nd	nd	nd	–	na	na	na	SIVmac 17E-Fr	nd	nd	252	26.8	17.6	210	30.7	31.0	284
	X70	nd	nd	nd		na	na	na		nd	nd	251	49.1	17.7	364	44.0	22.3	324
	X71	nd	nd	nd		na	na	na		nd	nd	260	33.7	22.5	261	35.7	26.6	299
	X72	nd	nd	nd		na	na	na		nd	nd	254	33.0	16.7	201	42.1	25.3	201

On the day of inoculation of SIVmac17E-Fr, 20 weeks after inoculation of the first virus, the mean proportion in group A of CD3^+^/CD4^+^ lymphocytes were 35.4% ± 8.6%, CD3^+^/CD8^+^ lymphocytes were 19.7% ± 7.7% and platelet levels 320 × 10^9^/L ± 70 × 10^9^/L. For group B the mean proportion of CD3^+^/CD4^+^ lymphocytes were 33.9% ± 5.6%, CD3^+^/CD8^+^ lymphocytes were 28.7% ± 3.2% and platelet levels 374 × 10^9^/L ± 61 × 10^9^/L.

At week 22 of the study the mean proportion in group A of CD3^+^/CD4^+^ lymphocytes were 34.1% ± 14.7%, CD3^+^/CD8^+^ lymphocytes were 20.1% ± 11.5% and platelet levels 374 × 10^9^/L ± 65 × 10^9^/L. For group B the mean proportion of CD3^+^/CD4^+^ lymphocytes were 39.8% ± 3.1%, CD3^+^/CD8^+^ lymphocytes were 28.1% ± 5.7% and platelet levels 382 × 10^9^/L ± 48 × 10^9^/L. For group C the mean proportion of CD3^+^/CD4^+^ lymphocytes were 35.7% ± 9.5%, CD3^+^/CD8^+^ lymphocytes were 18.6% ± 2.6% and platelet levels 259 × 10^9^/L ± 75 × 10^9^/L. At week 43 of the study, when all macaques were killed humanely, the mean proportion in group A of CD3^+^/CD4^+^ lymphocytes were 44.5% ± 8.2%, the mean proportion of CD3^+^/CD8^+^ lymphocytes were 19.4% ± 5.7% and platelet levels 324 × 10^9^/L ± 62 × 10^9^/L. For group B the mean proportion of CD3^+^/CD4^+^ lymphocytes were 33.8% ± 5.3%, the mean proportion of CD3^+^/CD8^+^ lymphocytes were 31.9% ± 10.8% and platelet levels 328 × 10^9^/L ± 179 × 10^9^/L. For group C the mean proportion of CD3^+^/CD4^+^ lymphocytes were 38.1% ± 6.1%, the mean proportion of CD3^+^/CD8^+^ lymphocytes were 26.3% ± 3.6% and platelet levels 277 × 10^9^/L ± 53 × 10^9^/L.

### Viral detection within the CNS

**Fig. 1:**
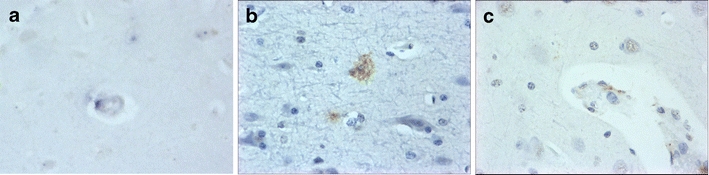
Representative images from frontal lobe showing results obtained following either SIVmacJ5 (**a**, **c**) or SIVmac17E-Fr (**b**) infection for **a** in situ hybridisation (×40), **b** anti-SIV env (×40) and **c** anti-SIV nef (×40)

SIV naïve macaques were negative for SIV by all viral detection methods used. SIV replication was detected in brain tissue by in situ hybridisation in all animals challenged with SIV (Table 3, Fig. 1). Immunohistochemical analyses detected the presence of SIV envelope (anti-gp41; KK41) in tissues from all SIV-infected macaques. However immunolabelling with the anti-SIV Nef antibody (KK75) detected signals only in macaques infected with SIVmacC8 and SIVmacJ5 (Table 3, Fig. 1).

**Table 3:** Viral replication in the different sections of the brain

	In situ hybridisation	*Env* (gp41)	nef Expression
Naïve Macaque			
Cerebrum	−ve	−ve	−ve
Midbrain	−ve	−ve	−ve
Brain Stem	−ve	−ve	−ve
Cerebellum	−ve	−ve	−ve
SIVmacC8			
Cerebrum	+^i^	+	+
Midbrain	+^i^	+	+
Brain Stem	+^i^	+	+
Cerebellum	+^i^	+	+
SIVmacJ5			
Cerebrum	+^i^	+	+
Midbrain	+^i^	+	+
Brain Stem	+^i^	+	+
Cerebellum	+^i^	+	+
SIVmac17E-Fr			
Cerebrum	+^i^	+	−ve
Midbrain	+^i^	+	−ve
Brain Stem	+^i^	+	−ve
Cerebellum	+^i^	+	−ve

Evidence of viral replication was shown by in situ hybridisation to detect viral RNA, gp41 (envelope protein) to show evidence of virion assembly, and *nef* expression to identify productively infected cells. Cerebrum = frontal, parietal, occipital, and temporal lobe sections; Midbrain = thalamus and midbrain sections; Brain stem = pons and medulla oblongata sections; Cerebellum = cerebellum. +^i^ ISH positive cells present between 0.5 and 6.8 + ve cells/mm^2^. + Immunoreactive cells identified in all sections examined. −ve No immunoreactive cells identified

### Pathological changes to the brain following SIV infection

**Fig. 2:**
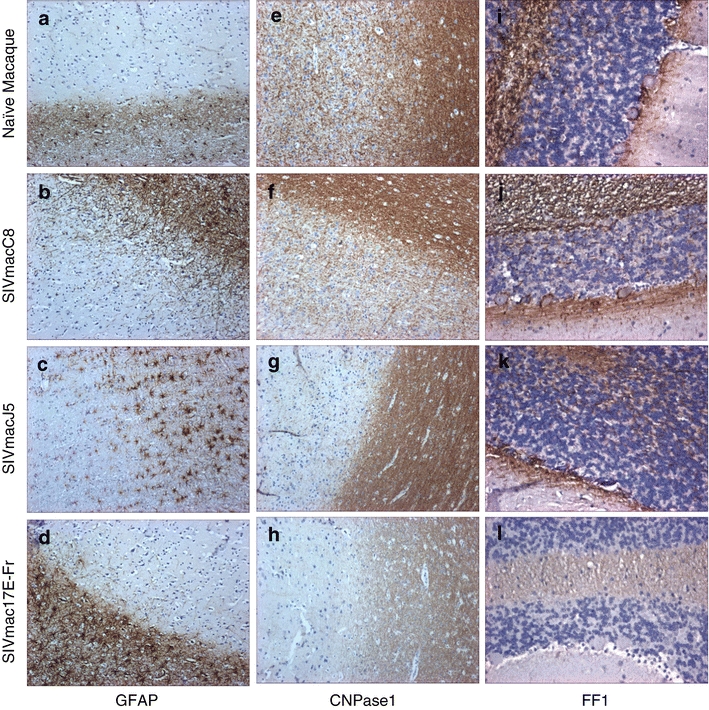
Representative images showing immunohistochemical staining results for **a**–**d** astrocytes (GFAP × 10), **e**–**h** oligodendrocytes (CNPase1 × 10) and **i**–**l** neuronal phosphorylation (FF-1 × 20) within **a**–**h** frontal lobe and **i**–**l** cerebellum of either SIV naive or SIV-infected brain samples

Brain sections of SIV naive macaques showed expression of GFAP (astrocytes), CNPase1 (oligodendrocytes) and FF1 (phosphorylated neurofilaments) across the white matter of all sections. Little alteration in expression levels of CNPase1 or FF1 was noted in brain sections from SIVmacC8-infected animals, whereas the intensity of staining for GFAP was markedly increased (Table 4, Fig. 2). Following infection with SIVmacJ5 or SIVmac17E-Fr there were alterations in expression levels of all three markers. Brain sections from SIVmacJ5-infected animals exhibited an increase in the intensity of GFAP expression, particularly in the cerebrum, midbrain and cerebellum. By contrast, CNPase1 and FF1 expression were reduced in these areas (Table 4, Fig. 2). Infection with SIVmac17E-Fr resulted in an increase in the intensity of GFAP staining (Table 4, Fig. 2), whereas CNPase1 and FF1 expression levels were greatly reduced across all tissue regions.

**Table 4:** Pathological changes to the macaque brain following infection with SIV

	Macrophage (CD68)	Microglia (iba-1)	Infected Microglia (CD163)	Astrocyte (GFAP)	Oligodendrocyte (CNPase1)	Neuron (FF1)
Naïve Macaque						
Cerebrum	+	+	–ve	+	+++	+++
Midbrain	+	+	–ve	+	+++	++
Brain Stem	+	+	–ve	+	+++	++
Cerebellum	–ve	+	–ve	+	+++	++
SIVmacC8						
Cerebrum	++	++	+	+++	+++	+++
Midbrain	+	++	+	+++	+++	++
Brain Stem	+	+++	−ve	+++	+++	++
Cerebellum	+	++	−ve	+++	+++	++
SIVmacJ5						
Cerebrum	+	+++	++	++	++	++
Midbrain	++	+++	+	++	++	+
Brain Stem	++	+++	+	+++	+++	++
Cerebellum	+	++	+	++	++	+
SIVmac17E-Fr						
Cerebrum	++	++++	++	++	+	+
Midbrain	+	++++	++	++	++	+
Brain Stem	++	++++	+	++	++	+
Cerebellum	++	+++	+	++	+	+

Macaques were infected with either SIVmacC8, SIVmacJ5, or SIVmac17E-Fr. Sections from the brain were examined for alterations to microglia, astrocytes, oligodendrocytes and neurones. Cerebrum = frontal, parietal, occipital, and temporal lobe sections; Midbrain = thalamus and midbrain sections; Brain stem = pons and medulla oblongata sections; Cerebellum = cerebellum. ++++ Very strong immunoreactive staining in all sections examined. +++ Strong immunoreactive staining in all sections examined. ++ Moderate immunoreactive staining in all sections examined. + Weak immunoreactive staining in all sections examined. –ve No immunoreactive staining identified

**Fig. 3:**
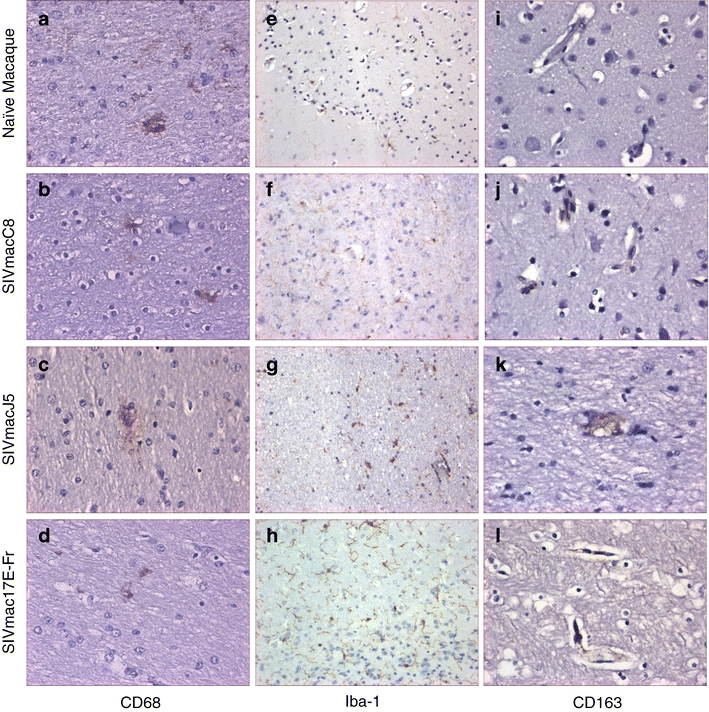
Representative images showing immunohistochemical staining results for **a**–**d** macrophage (CD68, ×40), E-H: microglia (iba-1, ×20), and **i**–**l** SIV-infected microglia (CD163, ×40) within frontal lobe of either SIV naive or SIV-infected brain samples

Analysis of the expression of the macrophage marker CD68 revealed an increased intensity of staining in all SIV-infected animals. The greatest increase was observed within the midbrain and brain stem of SIVmacJ5-infected macaques and cerebrum, brain stem and cerebellum of SIVmac17E-Fr-infected macaques (Table 4, Fig. 3).

Analysis of the expression of the microglial marker iba-1 revealed low numbers of pale stained cell bodies and processes in SIV naive animals (Table 4, Fig. 3). SIV-infected tissue exhibited cells where both round cell bodies and extended processes were more strongly stained. Expression levels of iba-1 were moderate in SIVmacC8-infected tissues in which cellular bodies with numerous cellular ramifications were stained. SIVmacJ5-infected tissues contained numerous strongly stained cellular bodies however the strongly stained cellular processes appeared truncated. The highest iba-1 expression levels were observed within SIVmac17E-Fr-infected tissues where large numbers of intensely stained cell bodies and processes were found. In addition, strongly stained amoeboid cells lacking any cellular processes were found scattered through the tissues (Table 4, Fig. 3).

Expression of the macrophage marker CD163 was not detected within brain tissue samples from SIV naive macaques. The cerebrum and midbrain of SIVmacC8-infected macaques exhibited low frequencies of CD163 positive cells associated with capillary vessels whereas this marker was detected with increasing frequency through SIVmacJ5 and SIVmac17E-Fr-infected tissues (Table 4, Fig. 3).

**Fig. 4:**
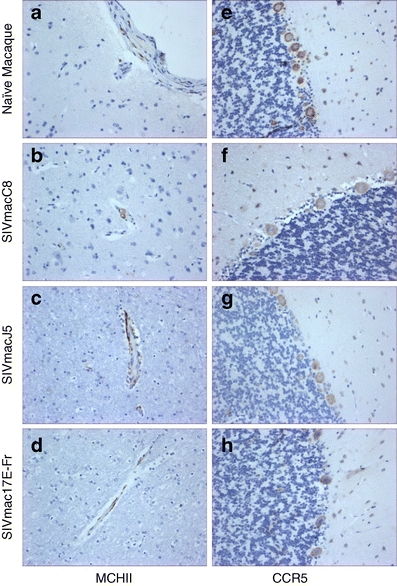
Representative images showing immunohistochemical staining results for **a**–**d** MHCII (×20) and **e**–**h** CCR5 (×20); **a**–**d** frontal lobe and **e**–**h** cerebellum of either SIV naive or SIV-infected brain samples

MHC class II expression was detected on small numbers of perivascular macrophage within all tissue sections from SIV naive macaques and all areas showed strong expression of the chemokine receptor CCR5, notably on neurones within the cerebral cortex and Purkinje cells within the cerebellum (Table 5, Fig. 4). Foci of increased MHCII expression within perivascular macrophage were observed following infection with either SIVmacJ5 or SIVmac17E-Fr. MHCII expression was not observed within migroglia. MHC class II expression levels in SIVmacC8-infected tissue were equivalent to those observed in SIV naive tissues (Table 5, Fig. 4). Following SIVmacC8 or SIVmacJ5 infection tissues showed an equal level of reduction of CCR5 expression in the midbrain, brain stem and cerebellum (Table 5, Fig. 4). Within SIVmac17E-Fr-infected tissues CCR5 expression levels were further reduced within the cerebrum and brain stem (Table 5).

**Table 5:** Immunological response to viral replication

	T-cell	MHC II	CCR5
Naïve Macaque			
Cerebrum	+ (CD3^+^)	+	+++
Midbrain	+ (CD3^+^)	+	+++
Brain Stem	+ (CD3^+^)	+	+++
Cerebellum	+ (CD3^+^)	+	+++
SIVmacC8			
Cerebrum	+ (CD3^+^, CD8^+^)	+	+++
Midbrain	+ (CD3^+^, CD8^+^)	+	++
Brain Stem	+ (CD3^+^)	+	++
Cerebellum	+ (CD3^+^, CD8^+^)	+	++
SIVmacJ5			
Cerebrum	+ (CD3^+^, CD4^+^, CD8^+^)	++	+++
Midbrain	+ (CD3^+^, CD8^+^)	++	++
Brain Stem	+ (CD3^+^)	++	++
Cerebellum	+ (CD3^+^, CD8^+^)	++	++
SIVmac17E-Fr			
Cerebrum	+ (CD3^+^, CD4^+^, CD8^+^)	++	+
Midbrain	+ (CD3^+^, CD8^+^)	++	++
Brain Stem	+ (CD3^+^)	++	+
Cerebellum	+ (CD3^+^, CD8^+^)	++	++

Macaques were infected with either SIVmacC8, SIVmacJ5, or SIVmac17E-Fr. Sections from the brain were examined for T-cell proliferation, MHC class II expression and CCR5 expression. Cerebrum = frontal, parietal, occipital, and temporal lobe sections; Midbrain = thalamus and midbrain sections; Brain stem = pons and medulla oblongata sections; Cerebellum = cerebellum. ++++ Very strong immunoreactive staining in all sections examined. +++ Strong immunoreactive staining in all sections examined. ++ Moderate immunoreactive staining in all sections examined. + Weak immunoreactive staining in all sections examined. −ve No immunoreactive staining identified

Low frequencies of CD3^+^ T cells were detected in brain tissues from all animals. There were no observable differences between the four groups (Table 5).

Similar levels of CD8^+^ T cells were detectable in the cerebrum, midbrain and cerebellum of all SIV-infected animals whereas CD4+ T cells were only detected in the cerebrum of SIVmacJ5 or SIVmac17E-Fr-infected animals (Table 5).

## Discussion

The initiation and development of the neuropathology that leads to either HAND or HAD remains unclear whilst the daily impact on patients and healthcare systems continues despite advances in ART (Ellis et al. 2007; Valcour et al. 2011; Letendre et al. 2010).

Accelerated models of SIV infection within rhesus macaques has been used extensively to model largely late stage HIV-associated pathology of the brain and central nervous system (Babas et al. 2003; Flaherty et al. 1997; Orandle et al. 2002: Overholser et al. 2003; Williams et al. 2001) By contrast, the CNS pathology induced following SIV infection of cynomolgus macaques (*Macaca fascicularis*) progressing at a natural rate has not been extensively described. The design of a vaccine study provided an opportunity to investigate the neuropathogenic potential of viruses with very different biological characteristics (Clarke et al. 2003; Berry et al. 2008) during a timeframe when peripheral levels of viral replication were minimal and there was no evidence of peripheral disease progression. Despite extensive analysis of PBMC and lymphoid tissues no evidence of SIVmac17E-Fr super infection was found within groups A and B enabling the neuropathology induced by the T cell tropic SIV clones SIVmacC8 and SIVmacJ5 to be determined. These results were analysed in the context of the increased rate of neuropathology development observed within cymomolgus macaques infected by the known neurotropic SIVmac17E-fr.

The development of neuropathology in cynomolgus macaques following SIV infection has been reported for a small number of animals only (Baskerville et al. 1990; Li et al. 1991; Mankowski et al. 1997; Zink et al. 1997) and the development of a severe neuropathology was not commonly found. This study extends this knowledge within a non-accelerated model for SIVmac17E-Fr (Flaherty et al. 1997) that has been used extensively in rhesus macaques because of its defined neurovirulence and SIVmacJ5 and SIVmacC8; molecular clones derived from the 32H re-isolate of SIVmac251. In vitro data indicates these clones are T cell tropic, (Rud et al. 1994). Furthermore, whilst SIVmacJ5 and SIVmacC8 are isogenic, SIVmacC8 expresses an attenuated phenotype in vivo as a result of a small in frame deletion in *nef* (Rud et al. 1994; Almond et al. 1995).

Despite SIVmac17E-Fr exhibiting a readily and effectively controlled primary viremia within cynomolgus macaques the detection of virus-infected cells within these brains was not unexpected due to the known neurotropic properties of SIVmac17E-Fr in rhesus and pig-tail macaques (Zink et al. 1997). There was less certainty whether SIVmacJ5 would be detected within this timeframe (Montgomery et al. 1998, 1999). Even more unexpected was the detection of the attenuated SIVmacC8 in the brain at a time when only low levels of virus were detected in the circulation by RT-PCR. If free virus is involved in neuro-invasion then, in cynomolgus macaques at least, it must occur during the early viremic phase. If this is the case it would indicate that clinical management of HIV neurological complications may need to take account of events that occur early after infection.

If, as it would appear, non neurotropic SIV is able to establish infection in neurological tissue relatively soon after infection, then where does it replicate? Many studies have identified cells of the monocyte macrophage lineage as being the major cell types infected following HIV or SIV infection of the brain (Albright et al. 2000; Kolson and Gonzalez-Scarano 2000; Clements et al. 2002; Cosenza et al. 2002; Gonzalez-Scarano et al. 2005).

The results using CD68 and CD163 indicate that following SIV infection expression levels are increased 43 weeks post SIVmacJ5 or SIVmacC8 challenge with the highest expression levels being found 23 weeks post SIVmac17E-Fr infection. Due to the morphology, tissue position, expression levels and disease state of the animals, it is likely that the cells expressing these markers are perivascular macrophage. CD163 staining of ramified activated microglia has currently only been reported in conjunction with SIV encephalitis (SIVE; Borda et al. 2008; Kim et al. 2006) and the likely activation, via CD163, following vascular leakage of Hp–Hb complexes through a damaged BBB. As a damaged BBB is a potential route of viral entry early in infection, it is unclear why microglial activation via CD163 is not present prior to the development of SIVE. A detailed examination of the timing of BBB disruption following SIV infection would provide further information for this debate.

The infiltration of T cells, especially CD8^+^ T cells, into the CNS following infection clearly indicates there had been disruption of the blood brain barrier at some point(s) during infection (Table 5). T cells are normally excluded from the brain, although they are known to cross the BBB during periods of neuroinflammation (Ballabh et al. 2004). SIV infection of rhesus macaques has been shown to disrupt lengths of the BBB (Luabeya et al. 2000; Maclean et al. 2005), as has HIV infection of humans (Annunziata 2003). Vascular leakage and subsequent inflammatory changes may explain the neuropathology identified. A longitudinal study with particular focus on the integrity of the BBB and inflammatory markers at various stages following SIV infection would provide further information regarding the aetiology and subsequent development of SIV associated neuropathology within cynomolgus macaques.

Microglial cells are considered to have an important role in SIV associated neuropathology. Within the brain, iba-1 has been shown to be expressed by resting microglia, with expression levels up-regulated following microglial activation (Imai et al. 1996). Levels of neuropathological iba-1 expression following SIV infection have not been extensively reported and, where they have, microglial expression levels have been shown to increase following CD8 depletion of SIVmac251-infected rhesus macaques (Ratai et al. 2010) and within microglia surrounding SIVE lesions in SIVmac239-infected rhesus (Borda et al. 2008). Similar to the results of Borda et al. (2008), we observed iba-1 staining of resting ramified microglia within brain tissue of SIV naive macaques.

Infection with either the *nef* attenuated SIVmacC8 or SIVmacJ5 increased iba-1 expression levels within microglial cell bodies and cytoplasmic processes with these processes being shortened in SIVmacJ5-infected tissue. Microglia within SIVmac17E-Fr-infected tissues expressed the highest levels of iba-1 notably also on microglia with an amoeboid morphology. The differential microglial morphologies observed may reflect transition between a ramified resting state and activated amoeboid state with this triggered most rapidly by neurotropic viral strains. That there are various stages of transition before full activation may be reflected in the lack of MHCII expression observed on microglia within infected tissues. MHCII expression was observed within an increasing numbers of foci of perivascular macrophage.

Previous studies have observed oligodendrocyte degradation and demyelination following SIV infection of rhesus macaques (Pope et al. 1997; Chretien et al. 2000; Marcario et al. 2004; Roberts 2005). This study found significant loss of CNPase1 expression within the white matter of cynomolgus macaques following SIVmac17E-Fr infection, with SIVmacJ5 infection reducing expression levels to a lesser extent. However following SIVmacC8 infection, CNPase1 expression levels were comparable with those observed in SIV naïve macaques further highlighting the differential pathology induced at this time point by a *nef* attenuated SIV.

Neurofilaments are highly phosphorylated structural proteins involved in the transfer of material from the cell body to the axonal foot (Lariviere and Julien 2004). HIV gp120 and tat induces neurofilament damage in tissue culture (Kaul and Lipton 1999; Ramirez et al. 2001) and within rhesus macaques infected with neurovirulent SIV (Adamson et al. 1996). This study demonstrates the ability of a neurovirulent SIV to alter neurofilament phosphorylation levels within cynomolgus macaques relatively early during the infection process and that this effect is also induced by wild-type lymphatropic SIV strains albeit at a slower rate. Neuronal effects of SIV infection were further observed by the loss of CCR5 expression by neuronal cell bodies within both the cerebrum and cerebellum. As SIV or HIV infection of neurones have not been demonstrated these neuronal effects may result from direct binding of viral proteins such as gp120 or Tat (Jones and Power 2006).

Whilst accelerated neurovirulent SIV/rhesus macaque models of HIV neuropathology provide a platform to study late stage disease relatively few studies have addressed its aetiology, notably early CNS invasion events and the subsequent pathologies that develop despite a controlled peripheral viremia. The availability of a model to investigate these areas is required to address many questions including what role persisting low level viral replication and/or viral protein/RNA expression plays in driving the development of neuropathology, can new therapeutic targets be identified, what are the neurological benefits of CNS penetrating therapies and how this information can be used to inform the debate regarding clinical use of early treatment for neurological benefit. Future studies investigating both tissue samples and matched CSF samples from this time frame could assist the identification of early peripheral markers of neuro-AIDS.

The changes observed within this study using standard neuropathology markers demonstrate that non-accelerated infections of cynomolgus macaques with SIV, including isolates not immediately recognised as neurotropic, can induce differential patterns of CNS pathologies. These are present despite limited peripheral viral replication or evidence of disease progression and develop at different rates. The range of pathologies observed potentially reflects different points on the spectrum of HIV clinical neuropathological patterns requiring treatment and healthcare support (Ellis et al. 2007; Lindl et al. 2010; Valcour et al. 2011).

Further studies of the early neuropathogenesis in cynomolgus macaques may provide information on a number of important questions such as when and how different virus strains enter the CNS and what tissue responses occur. Most important is the opportunity to use these simian models to define the role of ongoing virus replication on BBB integrity, inflammatory responses and subsequent pathological events. This will inform whether early intervention tailored to neurological events rather that treatment for late stage peripheral disease is beneficial in slowing or even stopping the development of a deleterious neuropathology.
